# Ischemic Stroke and Ruptured Mycotic Aneurysm, Two Complications of Infective Endocarditis in One Patient

**DOI:** 10.1155/2022/6275537

**Published:** 2022-09-19

**Authors:** Alassane Mamadou Diop, Ahmadou Bamba Mbodj, Serigne Abdou Aziz Fall, Ibrahima Niang

**Affiliations:** ^1^Cheikh Anta Diop University, Dakar, Senegal; ^2^Neurology Department of the Pikine Hospital, Dakar, Senegal; ^3^Neuroscience Department of the Fann Hospital, Dakar, Senegal; ^4^Medical Imaging Department of the Fann Hospital, Dakar, Senegal

## Abstract

The incidence of infective endocarditis is estimated to be around 30 cases per million inhabitants/year. It can be responsible for various neurological complications such as cerebral infarction, meningitis, cerebral abscesses, and cerebral hemorrhage due to ruptured mycotic aneurysms. Several germs have been incriminated in this condition including *Staphylococcus*, *Streptococcus*, and *Enterococcus*. We report the case of a 64-year-old patient who presented with an acute motor deficit of the left upper limb associated with dysarthria. MRI showed infarcts in both cerebral hemispheres, and the TOF sequence showed an amputation of M2. On transesophageal ultrasound, there was evidence of vegetations at the mitral valve. Blood culture isolated *Streptococcus oralis*. With antibiotic treatment, the evolution was marked by a stable apyrexia with regression of the dysarthria. Before her surgery, she suddenly developed aphasia with worsening of the motor deficit. CT scan showed a right fronto-parietal hematoma which was related to a ruptured cerebral aneurysm. She underwent endovascular embolisation and subsequent cardiac surgery.

## 1. Introduction

Infective endocarditis is responsible for neurological complications like ischemia, meningitis, brain abscess, and mycotic aneurysms, which are detected in 5% of patients in the acute and subacute phase of the infection and can be complicated by cerebral hemorrhage when ruptured [1]. Ischemic strokes due to vegetation embolism are considered the main neurological complication of endocarditis with two risk factors: the size and mobility of the vegetation [2]. Although recommendation of antibiotic therapy and surgical indications are now well defined in the recommendations, the ideal timing of surgery is still a matter of debate [3]. We report a case of streptococcal infective endocarditis complicated by ischemic stroke and cerebral hematoma due to a mycotic aneurysm rupture.

## 2. Observation

**Figure 1:**
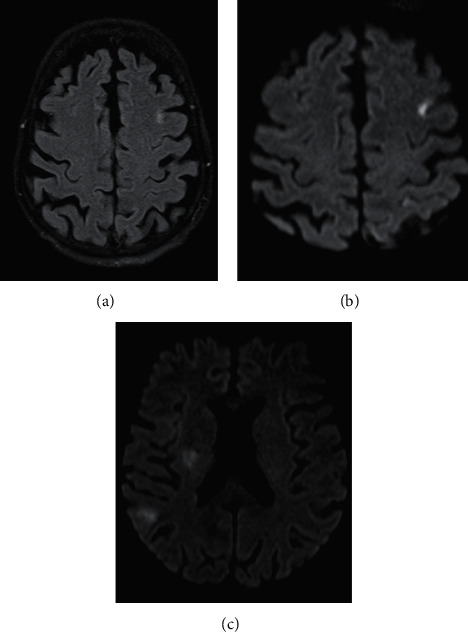
Bilateral ischemic lesions: at the left cortical frontal lobe in FLAIR and diffusion hypersignal (a, b) and in the right parietal and lenticular in diffusion hypersignal (c).

**Figure 2:**
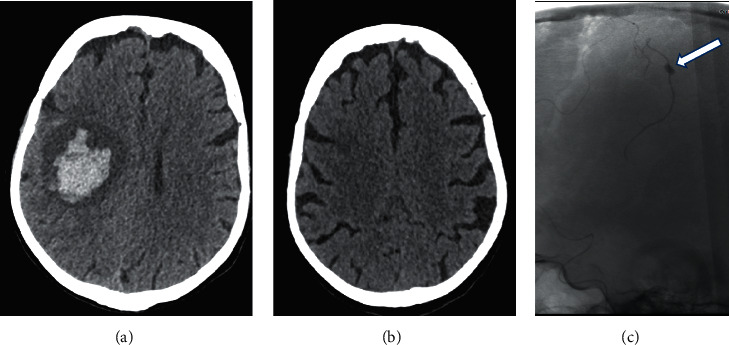
Hemorrhage from ruptured aneurysm right fronto-parietal hematoma (a); previously normal CT brain (b); angiography showing the aneurysm of a branch of the right middle cerebral artery (white arrow) (c).

The patient was 64 years old and was admitted to hospital with sudden onset of motor deficit of the left upper limb associated with speech disorders. The examination on admission revealed a left brachial monoplegia, dysarthria, a fever of 41 degrees Celsius, and a mitral focus murmur. Brain MRI in FLAIR and diffusion sequences showed subacute ischemic lesions in both cerebral hemispheres with a more significant lesion in the right internal capsule (Figure 1). On the 3D TOF sequence, we found an amputation in the M2 segment of the left sylvian artery. A transthoracic cardiac echocardiography performed in emergency suspected vegetations of the mitral valve. Transesophageal echocardiography showed Oslerian graft of both mitral leaflets with a small prolapse over a probable cord rupture and vegetation of 22 mm by 10 mm. The other valves and the myocardium were intact. The biology showed a CRP of 123 mg/l without hyperleukocytosis. Troponin and ProBNP were normal. Blood cultures were positive for *Streptococcus oralis*. The diagnosis of endocarditis complicated by stroke was retained and antibiotic therapy with penicillin at a dose of 4 million units every 6 hours was initiated at the beginning, followed by amoxicillin 2 g every 4 hours associated with preventive anticoagulation with 4000 IU of enoxaparin. The evolution was marked by apyrexia and disappearance of dysarthria. The patient was transferred to cardiology where a discussion for valve surgery was in progress with the surgeons when she presented with aphasia, headaches, and extension of the deficit to the lower limb giving a left hemiplegia. The brain scan showed a right fronto-parietal hematoma (Figure 2(a)) which was not present at admission (Figure 2(b)). Cerebral angiography revealed a probably mycotic aneurysm that ruptured, leading to this hematoma (Figure 2(c)). Endovascular embolisation of the aneurysm was performed. She was still aphasic and hemiplegic when she was transferred to cardiac surgery where a valve replacement was successfully performed.

## 3. Discussion

The incidence of infective endocarditis is estimated to be around 30 cases per million inhabitants per year in general population studies conducted in Western countries [4].

The diagnosis of endocarditis is based on a combination of clinical and microbiological criteria and additional examinations using the Duke criteria. Since the European recommendations in 2015, imaging is part of the diagnostic algorithm as a major criterion for endocarditis [3, 5]. *Staphylococcus* is now the most common organism causing infective endocarditis, followed by streptococci of oral origin and then enterococci [4]. Studies agree on the importance of finding the germ by repeated blood cultures to improve patient management [3]. Symptomatic ischemic stroke is reported in 10–35% of infective endocarditis and is the most common neurological complication: approximately 50% of neurological complications of infective endocarditis are strokes related to emboli from the vegetations [2, 6]. The pathogenesis of mycotic aneurysms results from the migration of septic emboli, which attach to the artery walls, leading to focal arteritis, necrosis, and pseudoaneurysm formation [7]. Our patient received preventive anticoagulation with enoxaparin at a dose of 4000 units per day. Elkaryoni et al. performed a large meta-analysis in the United States that included 41,545 patients with infective endocarditis, of whom 39,255 were not anticoagulated and 2290 were anticoagulated. After adjustment for potentially confounding variables, there was no significant difference in risk of death or embolic complications of IE with AC; however, risk of ICH was higher with use of AC but did not reach statistical significance. Our results do not support the use of AC in IE for reduction in risk of thromboembolic events [8].

Our patient could not benefit from thrombolysis due to the risk of hemorrhagic transformation and the ischemic lesions visible on the FLAIR sequence on cerebral MRI. In fact, during cerebral infarctions linked to infective endocarditis, thrombolysis multiplies the risk of intracranial hemorrhage by 4 and is therefore contraindicated. In contrast, thrombectomy has been shown to improve the functional prognosis of patients [3, 9].

Cerebral hemorrhage secondary to thrombolysis of cerebral ischemia has been described in patients with infective endocarditis. It is most often due to asymptomatic aneurysm rupture. Therefore, it is strongly recommended to perform CT angiography in addition to routine non-contrast head CT scan in patients with a history of infective endocarditis who present with acute neurologic symptoms, before they receive rtPA for acute ischemic stroke [10].

The main antibiotic treatment regimens according to the germs are defined in the ESC 2015 recommendations. For oral and bovis group streptococci, penicillin G 12–18 million·U/d intravenous (IV) in 4 or 6 injections or continuous administration, amoxicillin 100–200 mg/kg/d IV in 4 or 6 injections, or ceftriaxone 2 g/d IV in 1 injection can be used. The duration of treatment is 4 weeks for native valves and 6 weeks for prosthetic valves [3, 11].

The occurrence of cerebral hemorrhage due to aneurysm rupture in our patient delayed cardiac surgery. Patients with radiographic evidence of ischemic stroke from septic emboli can safely undergo valvular surgery for IE without increased risk of symptomatic hemorrhage; as for thrombolysis, it is recommended to perform CT angiography to detect aneurysms [2, 12].

Heparin is the drug of choice for the mandatory anticoagulation during cardiopulmonary bypass (CPB), but the optimal heparin dose during CPB is not known. Compared with a lower dose of heparin during CPB, a high dose of heparin had little effect on the point-of-care measurements of hemostasis. Higher heparin dose does not seem to offer benefit during CPB [13].

## 4. Conclusion

Neurological complications are often life-threatening and may cause sequelae. Moreover, they are likely to radically modify the management of patients. Indeed, their occurrence must raise the question of possible specific diagnostic or therapeutic procedures and that of the indication and delay of a possible cardiac surgery.

## Data Availability

No data were used to support this study.
